# Correction: Khan et al. Reducing the Invasiveness of Low- and High-Grade Endometrial Cancers in Both Primary Human Cancer Biopsies and Cell Lines by the Inhibition of Aquaporin-1 Channels. *Cancers* 2023, *15*, 4507

**DOI:** 10.3390/cancers18060996

**Published:** 2026-03-19

**Authors:** Sidra Khan, Noor A. Lokman, Martin K. Oehler, Carmela Ricciardelli, Andrea J. Yool

**Affiliations:** 1 School of Biomedicine, University of Adelaide, Adelaide, SA 5000, Australia; sidra.khan@adelaide.edu.au; 2 Adelaide Medical School, Robinson Research Institute, University of Adelaide, Adelaide, SA 5000, Australia; noor.lokman@adelaide.edu.au (N.A.L.); oehler.mk@gmail.com (M.K.O.); 3 Department of Gynaecological Oncology, Royal Adelaide Hospital, Adelaide, SA 5000, Australia

**Error in Figure**

In our published paper [1], a careful reader pointed out a mistake in Figure 4: “The effects of pharmacological agents (AQP channel inhibitors) on EC cell line invasion.” In panel D, the representative image used to illustrate the invasiveness of MFE-280 with resveratrol had been accidentally taken from a different treatment. The revised Figure 4 appears below, with the corrected panel marked by an asterisk. All authors have reviewed and approved this correction. The authors state that the scientific conclusions are unaffected. This correction was approved by the Academic Editor. The original publication has also been updated.

## Figures and Tables

**Figure 4:**
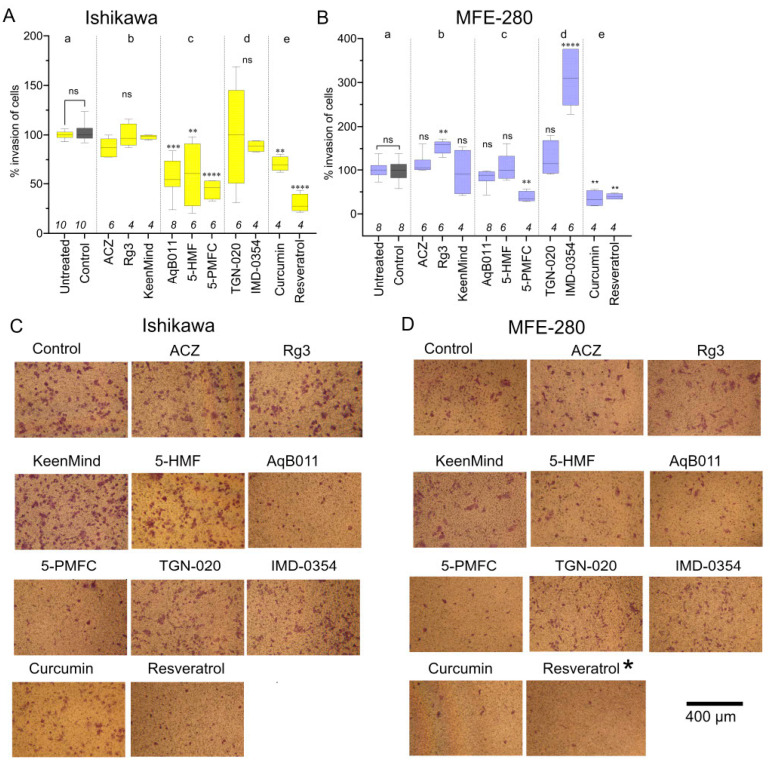
The effects of pharmacological agents (AQP channel inhibitors) on EC cell line invasion. Box plot summaries showing percent invasion of (**A**) Ishikawa after 24 h and (**B**) MFE-280 cells after 30 h treatments in (a) controls, or (b) with proposed AQP water channel inhibitors acetazolamide (ACZ, 1 µM for MFE-280, 10 µM for Ishikawa), ginsenoside Rg3 (100 µM), and KeenMind (44 µM); (c) AQP1 ion channel inhibitors 5-HMF (1.5 mM), AqB011 (60 µM for Ishikawa, 80 µM for MFE-280), 5-PMFC (0.5 mM); (d) proposed AQP water channel inhibitors TGN-020 (3 µM), IMD-0354 (0.2 µM); or (e) broad-spectrum inhibitors curcumin (20 µM) and resveratrol (40 µM). Statistical comparisons used one-way ANOVA and post hoc parametric *t*-tests. ** *p* < 0.01; **** *p* < 0.0001; *** *p* < 0.001; ns, not significant as compared with control. (**C**,**D**) Representative images of invaded cells for (**C**) Ishikawa (24 h) and (**D**) MFE-280 (30 h) with treatments as indicated (same doses as in (**A**,**B**)). Scale bar 400 µm. Asterisk (*) indicates the revised image for Resveratrol.
