# Supplementary material for: Reducing the Invasiveness of Low- and High-Grade Endometrial Cancers in Both Primary Human Cancer Biopsies and Cell Lines by the Inhibition of Aquaporin-1 Channels
Source: Cancers (Basel). 2023 Sep 11;15(18):4507. doi: 10.3390/cancers15184507 (PMC10526386; doi:10.3390/cancers15184507)
Supplement: Supplementary file 1 [file cancers-15-04507-s001.zip › cancers-2560170-supplementary.pdf]

List of Supplementary Materials

- Table S1. Patient data for endometrial cancer biopsy tissue samples.
- Fig S1. Epithelial cells of endometrial cancers stained positive for pancytokeratin.
- Fig S2. Morphology of epithelial cells compared to fibroblasts, visualized by light microscopy.
- Fig S3. AqB011 restraints the invasion of Ishikawa cells in 3D spheroid model
- Fig S4. Full membrane views of western blots for AQPs 1, 4, 8 and 11.
- Fig S5. Cell viability assay with pharmacological modulators of aquaporins and Traditional Chinese Medicines (TCMs)
- References cited

Table S1. Summary of diagnostic information for patient donors of endometrial cancer biopsy tissue samples. All samples were from patients formally diagnosed with endometrioid adenocarcinoma of the endometrium. Five samples were from grade 1 and three samples were from grade 3 endometrial cancer patients.

Patient ID number	Age at diagnosis	Stage	Grade
1	70	1a	1
2	67	1b	1
3	63	1b	1
4	67	1a	1
5	74	1b	1
6	68	2	3
7	70	3C1	3
8	68	1a	3

**Stage criteria (NIH National Cancer Institute, USA)*

Stage 1a: Cancer is in the endometrium only, or has invaded less than half of myometrium (muscle layer of the uterus).

Stage 1b: Cancer has spread into half or more of the myometrium.

Stage 2. Cancer has spread into cervix, but has not outside the uterus.

Stage 3C1: Cancer has spread to lymph nodes in the pelvis and/or around the aorta.

Grade 1 referred as low grade while grade 3 referred as high grade cancer.

Supplementary Figure S1

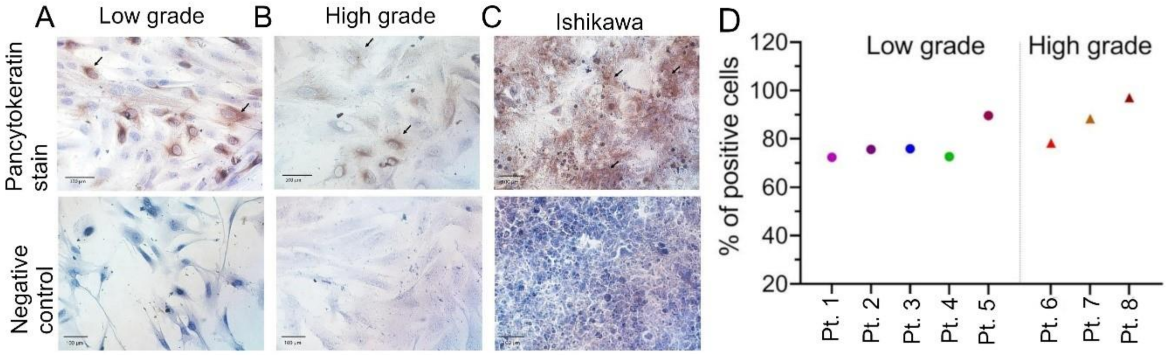

Figure S1. ***Epithelial cells of endometrial cancers stained positive for pancytokeratin.*** Positive pancytokeratin stain in (A) low grade and (B) high grade cancer tissues (top row) with respective negative controls (bottom row). Brown color shows the pancytokeratin staininining; cell nuclei are stained blue with haematoxylin counterstain. (C) The Ishikawa cell line was used as a postive control for pancytokeratin stain. (D) Summary of the percentage of positive cells, standardized to the total number of cells counted in six random microscopic fields of view. Scale bar 100 μm

Immunocytochemistry methods: Immunocytochemistry was done as described previously [1]. Briefly, Primary cancer cells were plated in Ibidi 8 well uncoated chamber slides (Cat #: 80841; Ibidi Germany) at 40,000 cells /ml (500 μl of cell suspension was added to each well). Briefly, at 80% confluency, fixation was done with ice cold methanol for 5 min, followed by ice cold acetone for 3 min. After two washes with ice cold PBS, peroxidase block was done with 0.3% H_2O_2 for 5 min. 5% goat serum (G9023-10ml) was used as a blocking agent followed by overnight incubation with primary antibody (1:750) at 4°C. The wells were incubated sequentially with biotinylated goat anti-mouse (E0432 1:400, Dako), followed by streptavidin-HRP conjugated (P0397, 1:500, Dako) for 1 hour at room temperature. Immunoreactivity was detected using diaminobenzidine/H_2O_2 substrate (Sigma-Aldrich). The sections were counterstained with 10% haematoxylin (Sigma-Aldrich). Imaging was done with an Olympus IX73 (Tokyo, Japan). Positive cells were counted manually by two researchers. Percentages of positive cells were calculated relative to total number of cells in six random fields. Ishikawa cells served as a positive control and mouse IgG (1 μg/ml) was added to negative controls instead of primary antibody.

Supplementary Figure S2

Figure S2. ***Morphology of epithelial cells in EC biopsy primary cultures as compared to fibroblasts, visualized by light microscopy.*** Images of primary EC cells from low grade (grade 1) (A) and High grade (grade 3) (B) EC tissues compared to fibroblasts (C). Epithelial cells cultured from different human endometrial cancer biopsies showed similar morphologies characterized by rounded or short spindle-shaped appearance that grew into colonies. In contrast, fibroblasts were more elongated flattened and densely aggregated. Scale bar 1000 μm.

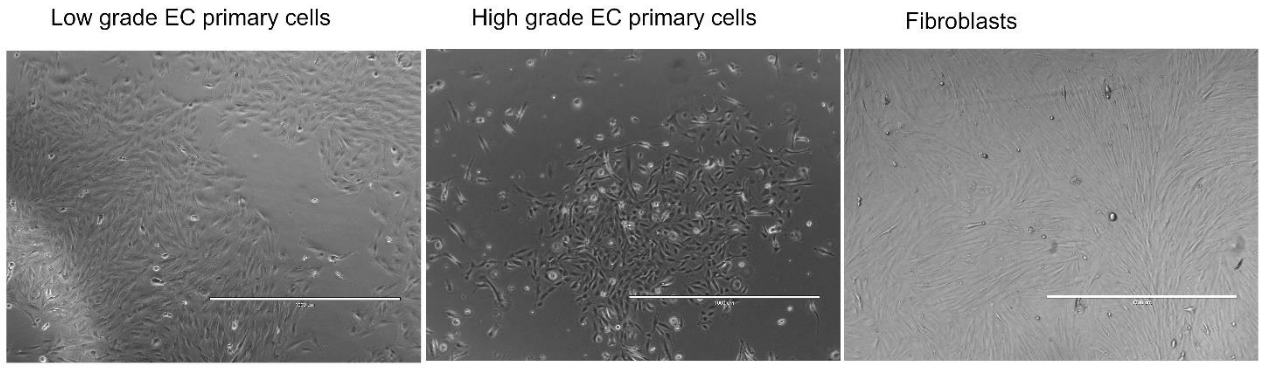

Supplementary Figure S3

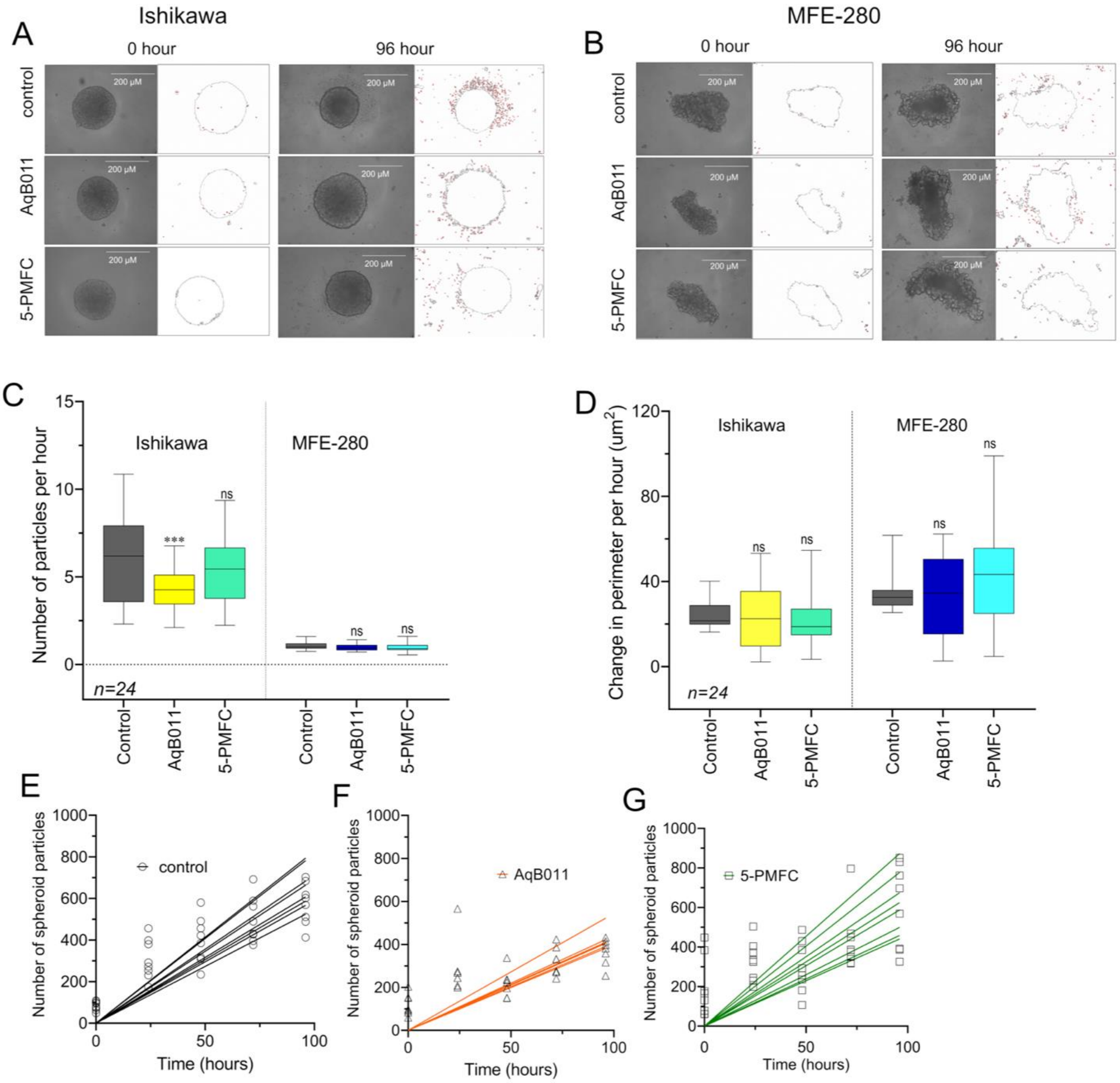

Figure S3. ***AqB011 restraints invasion of Ishikawa cells in 3D spheroid invasion model without compromising the viability of spheroids.*** Dispersal of cancer cells from solid tumor mass spheroids of Ishikawa and MFE-280 cells, with and without treatment with 100 µM AqB011 and 0.5mM 5-PMFC. (A,B) Representative images showing outlines of spheroids, surrounded by cancer cell clusters (particles) that disseminated into the surrounding matrigel, detected by NIH ImageJ at 0 and 96h of treatment with drugs or vehicle control. A box plot summary of cancer cell dispersal rates (C) as new particles per hour. (D) Box plots of rates of change in spheroid perimeter (µm/h) showed no differences in growth rates, with or without drug treatment in either cell line. Simple linear regressions of numbers of particles around spheroids of Ishikawa cell control group (black; E), AqB011 (orange; F) and 5-PMFC (green; G). Significant differences were analyzed by one way ANOVA followed by Mann Whitney tests with ****$p<0.0001$; ns not significant. Three independent experiments were done with 8 replicates each. Scale bar 100 µm.

Spheroid invasion assay methods:

Spheroid invasion assays were done using established protocols [2]. Briefly, polyHEMA (2-hydroxyethyl methacrylate, P3932-10G, Merck) coating solution was made (30mg/ml of polyhema

crystals dissolved in 95% ethanol), and shaken 48 h at 37 °C. 30 µl per well of polyhema was added to 96-well round bottom plates (Corn-ing-3799, NY, USA) in the dark, and kept under a sterile hood for 72 hrs. Plates were then exposed to UV light for 2 hrs prior to adding cells. Cell suspensions (5x103 cells/ml) of Ishikawa and MFE-280 cell lines in full DMEM were added at 100µl per well, and the plates were centrifuged at 600 rpm for 3 min followed by incubation for 48 h at 37 °C in 5% CO2. Spheroids for both cell lines were well aggregated by 48 h. Plates were cooled on ice for 15 min and 50 µl of 0.025 mg/ml Matrigel in DMEM was added to each well, followed by centrifugation (600 rpm at 4°C for 5 min) and incuba-tion 1 h to allow the Matrigel to set. At time zero, media with and without the indicat-ed test compound or an equivalent dose of vehicle were added. Time-lapse images were taken of the same spheroids at 0, 24, 48, 72 and 96 h using NIS-elements software (Olympus Phase Contrast ULWCD microscope; Notting Hill, VIC Australia). Parameters of spheroid area, perimeter, and the numbers of cells (particles) that had disseminated into the surrounding Matrigel environment were quantified using NIH Image J software (U.S. National Institutes of Health, Bethesda MD, USA; https://imagej.nih.gov/ij/download.html, last accessed Jan 2023). Rates of change were de-termined from data plotted as a function of time and fit with simple linear regression as per described previously [3]. Slope values (rates) for treatment groups were standardized to the mean rates for vehicle control groups. Three independent experiments were done with 8 to 9 replicates each.

Spheroid invasion assay results: Two AQP1 ion channel blockers AqB011 and 5-PMFC which were effective in transwell assays were tested in a spheroid model of invasion with Ishikawa and MFE-280 cells. Time lapse imaging was used to monitor the dissemination of clusters of cells (particles) from solid tumor masses (spheroids) into the surrounding Matrigel extracellular matrix (Fig S3 A, B). Incubation with 80 µM AqB011 over 96 h reduced rates of dissemination of Ishikawa cell clusters into the surrounding matrix as compared to control (Fig S3 C), without compromising cell viability measured by growth (Fig S3 D). Rates were calculated from linear regression fits of particle numbers plotted as a function of time (FigS3 E-G). Treatment with 0.5 mM 5-PMFC showed a trend towards decreased dispersion from Ishikawa spheroids. Low baseline rates of dispersion in MFE-280 prevented assessment of any inhibitory effects. Rates of particle dissemination measured as slope values (mean ± SD) for Ishikawa spheroids were 6.3 ±1.9 for vehicle control (Fig S3 D); 4.2 ±1.2 for AqB011 (Fig S3 E); and 5.4 ± 1.9 for 5-PMFC (Fig S3 F). Rates of particle dispersal from MFE-280 spheroids were low, averaging 1.1 ± 0.20 for vehicle control, 0.97 ± 0.19 with AqB011; and 0.96 ± 0.22 for 5-PMFC, perhaps suggesting a trend towards block but with signals too low to detect meaningful differences. There were no differences in the rates of increase in total perimeter of the spheroids over 0 to 96h between the control and pharmacological treatments (Fig S3 D), indicating that the AQP1 channel blockers were not indirectly impairing cell cluster dissemination by cytotoxicity.

Supplementary Figure S4

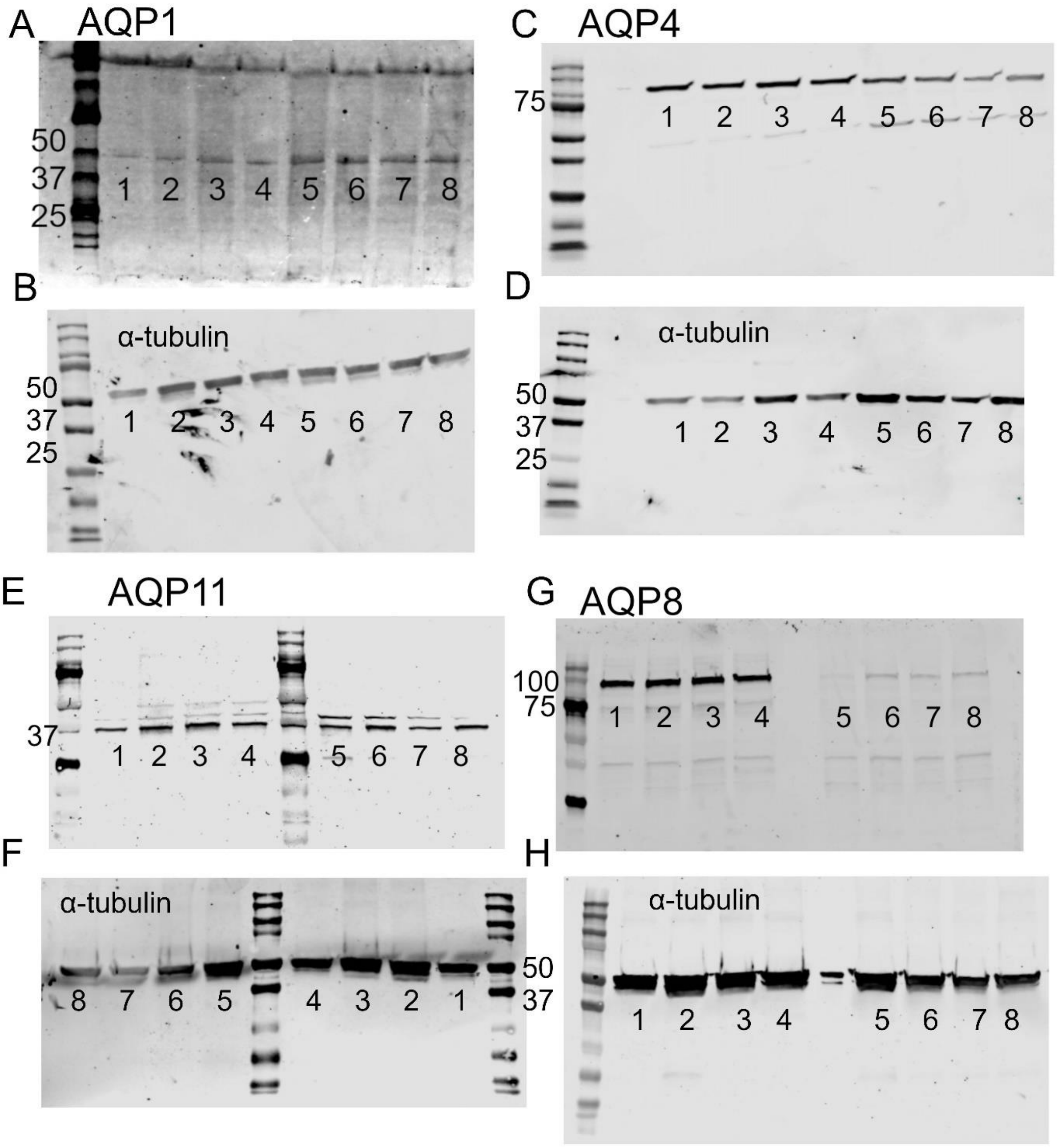

Figure S4. ***Full membrane views of western blots for AQPs 1, 4, 8 and 11.***

(A) **AQP1**= 28-35 kDa. From right to left, ladder, 1= Ishikawa untreated (band intensity relative to refrence protein= 0.161), 2= Ishikawa vehicle control, (0.1% ethanol), band intensity= 0.160, 3= Ishikawa 1 nM estradiol treated (band intesity, 0.35) , 4= Ishikawa 100 nM progesterone treated (band intensity=0.25). 5= MFE-280 untreated (band intensity=0.49), 6= MFE-280 vehicle control, band intensity=0.52 (0.1% ethanol), 7= MFE-280 1 nM estradiol treated (band intensity=0.67), 8= MFE-280 100 nM progesterone treated (band intensity=0.72). Reprobed with **(B)** α-tubulin= 55 kDa

(C) **AQP4**= 75 kDa. From left to right, ladder, 1= Ishikawa untreated (band intensity= 1.01) , 2= Ishikawa vehicle control (0.1% ethanol) band intensity=0.95, 3= Ishikawa 1 nM estradiol treated (band intensity=0.81), 4= Ishikawa 100 nM progesterone (band intensity=0.80). 5= MFE-280

untreated (band intensity, 0.42), 6= MFE-280 vehicle control (0.1% ethanol) band intensity= 0.44, 7= MFE-280 1 nM estradiol treated (band intensity, 0.32), 8= MFE-280 100 nM progesterone treated treated (band intensity= 0.46**)**. Reprobed with **(D)** α-tubulin= 55 kDa

(E) **AQP11**= 30-35 kDa. From left to right, ladder, 1= Ishikawa untreated (band intensity=0.24), 2= Ishikawa vehicle control (0.1% ethanol) band intensity=0.38 , 3= Ishikawa 1 nM estradiol treated (band intensity=0.346), 4= Ishikawa 100 nM progesterone treated (band intensity=0.31). 5= MFE-280 untreated (band intensity=0.199), 6= MFE-280 vehicle control, band intensity=0.29 (0.1% ethanol), 7= MFE-280 1 nM estradiol treated (band intensity=0.36), 8= MFE-280 100 nM progesterone treated (band intensity=0.33). Reprobed with **(F)** α-tubulin= 55 kDa

(G) **AQP8**= 100 kDa. From left to right, ladder, 1= Ishikawa untreated (band intensity, 0.43) , 2= Ishikawa vehicle control (0.1% ethanol) band intensity=0.46, 3= Ishikawa 1 nM estradiol treated (band intensity=0.49) , 4= Ishikawa 100 nM progesterone treated (band intensity= 0.44) 5= MFE-280 untreated (band intensity=0.027), 6= MFE-280 vehicle control, band intensity=0.057 (0.1% ethanol), 7= MFE-280 1 nM estradiol treated (band intensity=0.053), 8= MFE-280 100 nM progesterone (band intensity=0.039). Reprobed with **(H)** α-tubulin= 55 kDa

Supplementary Figure 5

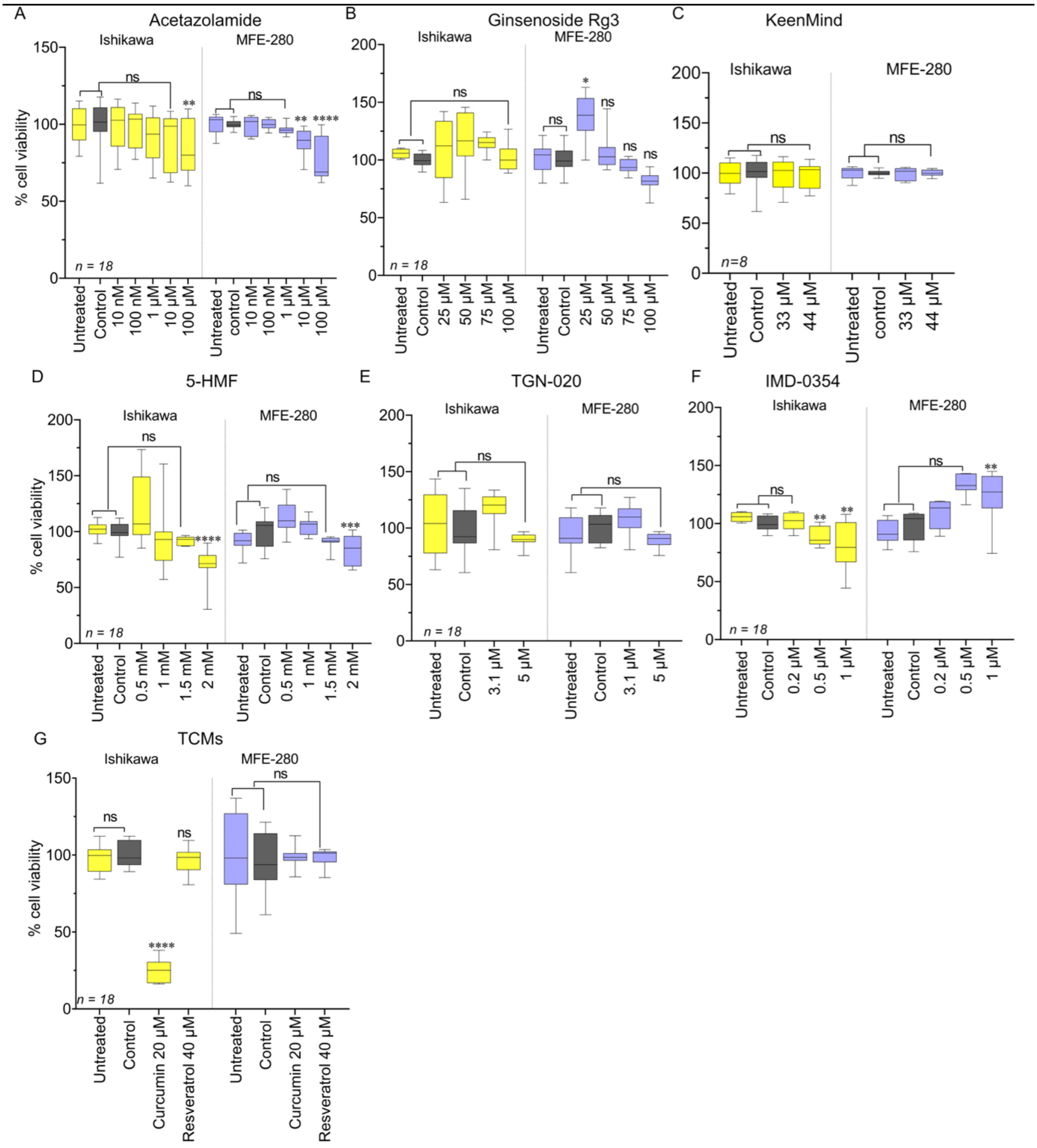

Figure S5. ***Evaluation of cytotoxicity of pharmacological modulators of aquaporins in EC cell lines.*** Viability of Ishikawa and MFE-280 cells after 24 h treatments with (A) Acetazolamide (10 nM-100 µM) , (B) Ginsenoside Rg3 (25 µM-100 µM) , (C) KeenMind (33 µM-44 µM), (D) 5-HMF (0.5 mM-2 mM), (E)TGN-020 (3.1 µM, 5 µM), (F) IMD-0354 (0.2 µM-1 µM)and (G) Curcumin (20 µM) and Resveratrol (40 µM), compared with matched untreated and vehicle control groups. 3 independent experiments were done with 6 replicates each (shown as n=18). Statistical analyses used one way ANOVA with post hoc parametric T tests, with: * $p<0.05$; ** $p<0.01$; and NS not significant).

Cell viability assay results: Cytotoxic effects of agents used in motility assays were measured by mitochondrial metabolic activity in the MTT assay. Effects of pharmacological modulators at 24 h on viability of Ishikawa and MFE-280 cells are summarized in Figure S5. In most treatments, toxicity either was not observed or became evident only at high doses, as seen for acetazolamide (Fig S5 A) which was toxic at doses 100-fold higher than those used in transwell assays. Ginsenoside Rg3, KeenMind, TGN-

020, and resveratrol (Fig S5 B,C,E, G) were not toxic in either cell line. Surprisingly, IMD-0354 and ginsenoside Rg3 increased mitochondrial metabolic activity in MFE-280 (Fig S5 B,F), and unexpectedly boosted MFE-280 transwell invasiveness (Fig. 4 main text); further work is needed to determine whether these effects are linked. Curcumin (Fig S5 G) proved to be toxic to Ishikawa cells, consistent with prior studies [4,5]. Resveratrol (Fig S5 G), which among many mechanisms of action is thought to downregulate AQP4 expression [6] and inhibit ion channels [7-9] was non toxic in both cell lines at effective anti-invasive doses, showing promising therapeutic potential.

References Cited

1. Ricciardelli, C.; Lokman, N.A.; Pyragius, C.E.; Ween, M.P.; Macpherson, A.M.; Ruszkiewicz, A.; Hoffmann, P.; Oehler, M.K. Keratin 5 overexpression is associated with serous ovarian cancer recurrence and chemotherapy resistance. *Oncotarget* **2017**, *8*, 17819.
2. Nakhjavani, M.; Palethorpe, H.M.; Tomita, Y.; Smith, E.; Price, T.J.; Yool, A.J.; Pei, J.V.; Townsend, A.R.; Hardingham, J.E. Stereoselective anti-cancer activities of ginsenoside Rg3 on triple negative breast cancer cell models. *Pharmaceuticals* **2019**, *12*, 117.
3. Varricchio, A.; Khan, S.; Price, Z.K.; Davis, R.A.; Ramesh, S.A.; Yool, A.J. Pharmacological Inhibition of Membrane Signaling Mechanisms Reduces the Invasiveness of U87-MG and U251-MG Glioblastoma Cells In Vitro. *Cancers (Basel)* **2023**, *15*, doi:10.3390/cancers15041027.
4. Sirohi, V.K.; Popli, P.; Sankhwar, P.; Kaushal, J.B.; Gupta, K.; Manohar, M.; Dwivedi, A. Curcumin exhibits anti-tumor effect and attenuates cellular migration via Slit-2 mediated down-regulation of SDF-1 and CXCR4 in endometrial adenocarcinoma cells. *The Journal of Nutritional Biochemistry* **2017**, *44*, 60-70.
5. Chen, Q.; Gao, Q.; Chen, K.; Wang, Y.; Chen, L.; Li, X. Curcumin suppresses migration and invasion of human endometrial carcinoma cells. *Oncology letters* **2015**, *10*, 1297-1302.
6. Li, W.; Tan, C.; Liu, Y.; Liu, X.; Wang, X.; Gui, Y.; Qin, L.; Deng, F.; Yu, Z.; Hu, C. Resveratrol ameliorates oxidative stress and inhibits aquaporin 4 expression following rat cerebral ischemia-reperfusion injury. *Molecular medicine reports* **2015**, *12*, 7756-7762.
7. Lee, B.-H.; Choi, S.-H.; Hwang, S.-H.; Kim, H.-J.; Lee, J.-H.; Nah, S.-Y. Resveratrol inhibits GABACρ receptor-mediated ion currents expressed in xenopus oocytes. *Korean J Physiol Pharmacol* **2013**, *17*, 175-180.
8. Vancauwenberghe, E.; Noyer, L.; Derouiche, S.; Lemonnier, L.; Gosset, P.; Sadofsky, L.R.; Mariot, P.; Warnier, M.; Bokhobza, A.; Slomianny, C. Activation of mutated TRPA1 ion channel by resveratrol in human prostate cancer associated fibroblasts (CAF). *Molecular carcinogenesis* **2017**, *56*, 1851-1867.
9. Wei, S.; Liu, T.T.; Hu, W.P.; Qiu, C.Y. Resveratrol inhibits the activity of acid‐sensing ion channels in male rat dorsal root ganglion neurons. *Journal of Neuroscience Research* **2022**.
